# The Roles of Human DNA Methyltransferases and Their Isoforms in Shaping the Epigenome

**DOI:** 10.3390/genes10020172

**Published:** 2019-02-23

**Authors:** Hemant Gujar, Daniel J. Weisenberger, Gangning Liang

**Affiliations:** 1 Department of Urology, Keck School of Medicine, University of Southern California, Los Angeles, CA 90033, USA; gujar@usc.edu; 2 Department of Biochemistry & Molecular Medicine, Keck School of Medicine, University of Southern California, Los Angeles, CA 90033, USA; dan.weisenberger@med.usc.edu

**Keywords:** DNA methylation, DNA methyltransferase, DNMT, DNMT3B, epigenetics

## Abstract

A DNA sequence is the hard copy of the human genome and it is a driving force in determining the physiological processes in an organism. Concurrently, the chemical modification of the genome and its related histone proteins is dynamically involved in regulating physiological processes and diseases, which overall constitutes the epigenome network. Among the various forms of epigenetic modifications, DNA methylation at the C-5 position of cytosine in the cytosine–guanine (CpG) dinucleotide is one of the most well studied epigenetic modifications. DNA methyltransferases (DNMTs) are a family of enzymes involved in generating and maintaining CpG methylation across the genome. In mammalian systems, DNA methylation is performed by DNMT1 and DNMT3s (DNMT3A and 3B). DNMT1 is predominantly involved in the maintenance of DNA methylation during cell division, while DNMT3s are involved in establishing *de novo* cytosine methylation and maintenance in both embryonic and somatic cells. In general, all DNMTs require accessory proteins, such as ubiquitin-like containing plant homeodomain (PHD) and really interesting new gene (RING) finger domain 1 (UHRF1) or DNMT3-like (DNMT3L), for their biological function. This review mainly focuses on the role of DNMT3B and its isoforms in *de novo* methylation and maintenance of DNA methylation, especially with respect to their role as an accessory protein.

## 1. Introduction 

With the improvement in DNA-sequencing technologies, genetic alterations were initially recognized as the main contributors of the initiation and progression of diseases including cancer [1,2,3,4]. However, epigenetic mechanisms are becoming increasingly recognized as the drivers of tumorigenesis and tumor progression [5,6,7,8]. The epigenome of an organism represents the collection of cellular processes that alter gene regulation, which are not attributed to the DNA sequence alone. This information is represented by histone modification, histone variants, nucleosome occupancy, expression of non-coding regulatory RNAs, and DNA methylation. These modifications dynamically regulate gene expression to maintain physiological processes, cellular function, and organismal development [5,7,9,10,11,12,13,14,15,16,17,18,19].

### 1.1. Nucleosomal Structure and Histone Modifications 

Epigenetic and genetic factors interplay to maintain physiological states of cells and are often altered and dysregulated across virtually every form of human cancer [5,6,20,21]. The genome in a cell is tightly packaged around histone proteins to form a basic nucleosome structure [19,22]. Specifically, the nucleosome consists of 145–147 bp of DNA wrapped around a core histone (H) octamer that is composed of two copies each of H2A, H2B, H3, and H4 proteins with a 10–70-bp linker DNA and linker histone H1 attached. As a result, the nucleosomal structure can physically restrict the access of proteins with DNA. Nucleosome occupancy at any given genomic location is defined as the cell population fraction occupied by histones, and may be effected due to various factors such as DNA motifs, DNA methylation, and histone modifications [23]. In general, active functional elements, such as promoters and enhancers, are free of nucleosomes in an open structure often referred to as a nucleosome-depleted region (NDR). 

Chemical modifications to chromatin such as histone acetylation, methylation, phosphorylation, ubiquitinylation, sumoylation, adenosine diphosphate (ADP) ribosylation, deamination, propionylation, and butyrylation are known to influence the interaction of histones with non-histone proteins, as well as overall chromatin structure [24] and protein accessibility, which in turn influence chromatin accessibility and regulate gene expression [19,22]. For example, histone H3 lysine 4 trimethylation (H3K4me3) occupancy in gene promoter regions and histone H3 lysine 36 trimethylation (H3K36me3) in gene body regions are both associated with actively transcribed genes, while histone H3 lysine 27 trimethylation (H3K27me3) at gene promoters is associated with suppressed gene expression [22]. Histone reader enzymes scan the nucleosomal topography and structure for recognizing specific histone modifications. Histone tail marks are placed by specific enzymes (writers) and are removed by enzymes known as erasers. A comprehensive list of histone erasers, writers, and readers and the roles of specific histone tail modifications was previously described [6,22,25,26].

The highly conserved core histones are replaced by variant versions as a result of DNA replication and DNA repair mechanisms. Histone variants are expressed throughout the cell cycle with a unique copy number in the genome, and show varying interaction with chromatin modifiers or may simply act as replacements for core histones [27]. The fundamental chromatin structure nucleosome is also regulated by adenosine triphosphate (ATP)-dependent chromatin-remodeling complexes that regulate DNA packaging in the nucleus and, thus, affect the accessibility of various factors to specific DNA regions to regulate gene expression, replication, repair, and recombination [28]. 

### 1.2. Non-Coding RNAs

Non-coding RNAs also have important roles in regulating gene expression, signaling networks, and the epigenome [29]. Non-coding RNAs are divided in two groups: small RNAs and long non-coding RNAs (lncRNAs). Small RNAs include microRNAs (miRNAs), small interfering RNAs (siRNAs), and piwi-interacting RNAs (piRNAs), and regulate gene expression through the canonical RNA interference (RNAi) pathway [29,30]. The function of piRNAs is in concert with the piwi protein Aubergine to silence repetitive elements during germ cell development, most notably in *Drosophila* and mouse systems [30,31,32]. Piwi-interacting RNAs may also act as cancer biomarkers, and P-element-induced wimpy testis (PIWI) proteins are upregulated in several cancer types as compared to normal cells [33]. Long ncRNAs have roles in transcriptional activation and repression, recruiting lineage-specific complexes, serving as scaffolds, regulating RNA splicing, and sequestering miRNAs [34,35]. A well-known lncRNA is *X inactive specific transcript* (*XIST*), RNA that interacts with several proteins to silence the X-chromosome [36]. *XIST* also mediates localization of the H3K27me3 repressive histone mark though the polycomb repressive complex 2 (PRC2) and Jumonji and AT-rich interaction domain containing 2 (JARID2) [37]. 

### 1.3. DNA Methylation

Among the various DNA modifications, 5-methylcytosine (5mC) in the 5’-CpG-3’ is common and well documented in mammalian genomes [38,39]. DNA methylation influences cellular differentiation, physiological conditions, X-chromosome inactivation [40,41], gene imprinting [42], and repression of retrotransposons [43,44]. DNA methylation profiles are stably inherited in mitotically dividing cells and are conserved with respect to the cell type. During development, DNA demethylation and remethylation occur in the migratory and post-migratory primordial germ cells, as well as in pre-implantation and early post-implantation stages [45].

Cytosine methylation is inherently mutagenic, as DNA methylation drives mutation rates that occur in cancers and other diseases [5,6]. Indeed, 5-methylcytosine is prone to spontaneous deamination to thymine; thus, the CpG sequence context is reduced to approximately 20% of what is predicted in the human genome. Furthermore, 5mC deamination also results in the creation of guanine–thymine mismatches that are repaired in an error-prone fashion, while cytosine-to-uracil deamination is easily flagged for repair, as uracil is not part of the DNA genome code. Methylated cytosines occur predominantly in CpG-poor regions of the genome and at repetitive elements; however, CpG-dense regions, termed CpG islands, have the expected CpG content and are usually devoid of methylation in normal somatic cells [46]. 

In gene promoter regions, DNA methylation can affect gene expression by regulating the recruitment of methylated DNA binding proteins (MBDs), which influence transcription factor binding and overall chromatin structure [47,48]. Specifically, DNA methylation blocks the interaction of some transcriptional activators with DNA sites or allow binding of repressive factors and insulators containing methyl-CpG-binding proteins to repress transcription [49,50]. In addition, genes marked with H3K27me3 are maintained in the differentiating cells in the presence of EZH2, an H3K27me3 methyltransferase, a component of the PRC2. In cancer cells, this complex recruits DNA methyltransferases (DNMTs) to establish DNA methylation patterns [51]. 

Concurrently, DNA methylation in gene bodies is associated with actively transcribed genes [48,52,53] and regulation of splicing [54,55]. DNA methylation at transcribed regions stabilizes the nucleosome for efficient RNA polymerase II (Pol II) transcriptional elongation, inhibits spurious transcription, represses retrotransposon activation, and helps facilitate RNA splicing [53,56,57]. In addition, gene body DNA methylation in actively transcribed genes is also associated with the active H3K36me3 histone mark that interacts with elongation factors for high transcription activity [58]. 

### 1.4. DNA Methylation Aberrancies in Human Cancers

Genes associated with regulation of the epigenome are often mutated in several tumor types [23]. Epigenetic gene regulation is thought to be a main and early driver in tumorigenesis, especially global DNA hypomethylation and gene promoter DNA hypermethylation [59]. For example, DNA hypermethylation in the promoter regions of the *SFRP* (secreted frizzled-related proteins) tumor suppressor gene family, inhibitors of the WNT signaling pathway, complements the effect of downstream mutations in human colorectal cancer [60]. In addition, *HIC1* (hypermethylated in cancer 1) promoter DNA hypermethylation is an early event in several cancers such as epithelial cancers in males and lymphomas and sarcomas in females [61]. Moreover, promoter DNA hypermethylation of genes involved in DNA repair, such as *MLH1*, *BRCA1*, *BRCA2*, and *XRCC5* correlates with reduced expression and results in enrichment of carcinogenic mutations in several forms of human cancers [62]. 

### 1.5. DNA Demethylation

DNA methylation is reversed by the ten-eleven translocase (TET) group of enzymes using vitamin C as a co-factor. TET enzymes oxidize 5-methyl-cytosine to cytosine through a 5-hydroxymethyl-cytosine (5-hmC) intermediate. The oxidation of 5-hmC generates 5-formylcytosine and 5-carboxycytosine, which are then replaced with unmethylated cytosine through the base excision repair pathway [63]. TET proteins have a common *C*-terminal catalytic domain. TET1 and TET3 have an additional CXXC domain which may facilitate binding to DNA [63]. TET enzymes are found at a higher concentration at transcription start sites of CpG island promoter regions [64]. TET mutations were reported in several solid tumors and myeloid tumors where their loss of function might promote aberrant DNA methylation [65]. TET2 mutations affecting enzyme function were reported in myeloid disorders, whereas TET1 and TET3 mutations were also in hematopoietic malignancies [63]. In addition, low levels of 5-hmC were observed in various cancer types, suggesting that low TET activity contributes to tumorigenesis and cancer progression [66]. 

## 2. DNA Methyltransferases (DNMTs)

DNA methylation is catalyzed by a conserved set of enzymes, termed DNMTs. DNMTs contain an *N*-terminal regulatory domain and a *C*-terminal catalytic domain, and include DNMT1, DNMT3A, DNMT3B, and Dnmt3c. The structure, mechanism, and function of DNMTs were previously described in detail [67,68,69,70,71]. An unusual DNMT reported is DNMT2, which consists of only a catalytic domain. Subsequently, DNMT2 was shown to lack DNA methyltransferase activity and functions instead as a transfer RNA (tRNA) methyltransferase [72]. DNMTs are highly expressed during DNA replication in the synthesis S-phase of the cell cycle, as well as in embryonic, somatic, and cancer cell types [73]. 

DNMTs function in maintenance and *de novo* functions, with the DNA methylation profiles generally maintained after DNA replication and cell division by DNMT1, while DNMT3A and DNMT3B are largely involved in facilitating *de novo* DNA methylation profiles at loci that were previously unmethylated [74]. However, it should be noted that DNMT1, DNMT3A, and DNMT3B function together in maintaining DNA methylation during DNA replication. In vitro studies also indicated a role of Dnmt1 in *de novo* methylation maintenance succeeding the Dnmt3 activity in murine systems [75]. DNMT1 is the primary methyltransferase its activity is sufficient to methylate DNA in CpG-poor regions [76,77]. However, CpG-dense regions require cooperativity with DNMT3A/3B to maintain DNA methylation. In addition, repetitive elements require both Dnmt1 and Dnmt3a and/or Dnmt3b for maintenance of DNA methylation in mouse embryonic stem cells [76]. 

**Figure 1:**
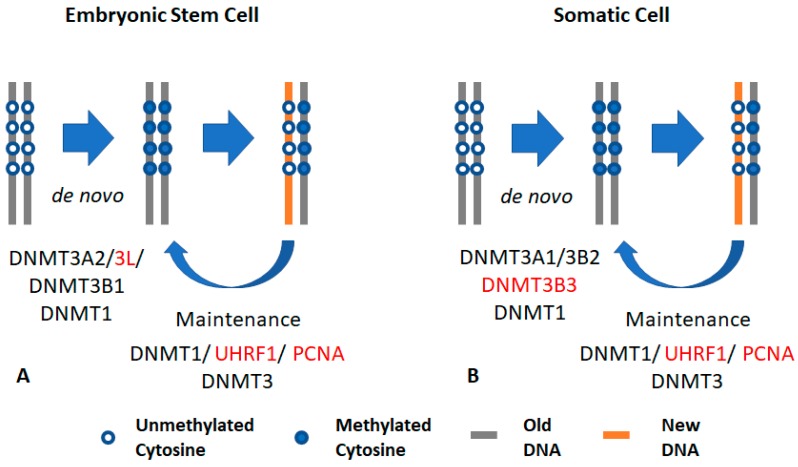
General concept of DNA methylation: (**A**) DNA methylation in embryonic stem cells (ESCs): ESCs express DNA methyltransferases 3A2 and 3L (DNMT3A2 and DNMT3L), which interact with each other through the carboxy domain. DNMT3L interacts with the unmethylated form of histone H3K4. The catalytically active isoform DNMT3B1 is also highly expressed in embryonic cells. UHRF1 and DNMT1 also maintain DNA methylation in ESCs. (**B**) DNA methylation in somatic cells: somatic cells mainly express and maintain methylation using DNMT1 and its accessory protein UHRF1. Somatic cells express the catalytically active DNMT3A1 and DNMT3B2 (at low expression levels) that interact with DNMT3B isoforms to catalyze methyl transfer. This interaction is required in the maintenance process. Somatic cells also express the catalytically inactive isoform, DNMT3B3. The black font indicates active DNMT, while the red font indicates an accessary protein.

DNMT3A and DNMT3B are mainly expressed in undifferentiated cells, where they are essential for the formation and subsequent maintenance of DNA methylation marks [15,76,78,79]. Both DNMTs are expressed in several isoforms that are essential during embryonic development and are dysregulated in human cancers [80,81,82,83,84]. Although DNMT1 is a key enzyme for maintenance of DNA methylation, the double knockout of *Dnmt3a* and *Dnmt3b* in mouse embryonic cells resulted in a gradual loss of DNA methylation over time, indicating the involvement of Dnmt3a/Dnmt3b in maintaining DNA methylation profiles during embryonic development (Figure 1) [76,77]. 

In general, DNMT3A or DNMT3B have roles in hematopoietic stem cell (HSC) self-renewal, maturation, and differentiation [85]. Knocking out *Dnmt3b* in mouse embryonic cells revealed its roles in bone mineralization, normal limb development, and bone morphogenetic protein signaling [86], while Dnmt3a affects bone density by effecting osteoclast differentiation [87]. Dnmt3b is also required postnatally in mice for fracture repair and angiogenesis [88], and human DNMT3B mutations are known to cause immunodeficiency, centromeric instability, and facial anomalies (ICF) syndrome [83], in which loss of DNMT3B activity results in DNA hypomethylation of the centromeric regions [89], as well as reduced interaction with the DNMT3L accessory protein [81]. 

### 2.1. DNMT1

DNMT1 is mainly responsible for the maintenance of DNA methylation and performs this task by copying the DNA methylation patterns from the parental DNA strand to the daughter DNA strand in coordination with DNA replication and cell division. DNMT1 consists of a *C*-terminal catalytic domain and an *N*-terminal regulatory domain. Interestingly, the DNMT1 *C*-terminal domain consists of two sub-domains: the methyltransferase domain and the target-recognition domain (TRD) responsible for recognizing hemimethylated cytosines [90]. The regulatory domain contains a DNA methyltransferase 1-associated protein 1 (DMAP1) binding domain, a replication foci targeting sequence (RFTS) domain, a CXXC domain, and a tandem bromo-adjacent homology (BAH1/2) domain [91]. *De novo* activity of DNMT1 is inhibited by the BAH domain, which positions itself between the unmethylated CpG-binding CXXC domain and the catalytic site [70]. Alternatively, the RFTS and CXXC domains may also interact with each other to inhibit DNMT1 access to DNA [92].

The high fidelity of DNMT1 toward hemimethylated DNA is maintained by both self-regulation of DNMT1 activity and by the accessory protein UHRF1 [93]. UHRF1 binds to DNA using its methyl DNA binding domain SRA (SET and RING associated), which preferentially binds to hemimethylated DNA [93]. UHRF1 directly recruits DNMT1 to hemimethylated DNA by removing the auto-inhibitory mechanism of the RFTS domain [71,92]. Interaction of proliferating cell nuclear antigen (PCNA), an accessory protein in DNA replication and repair, also reportedly interacts with DNMT1 [94]. In mouse embryonic stem cells (ESCs), the interaction of PCNA with DNMT1 enhances the efficiency and fidelity of the maintenance of DNA methylation [95]. 

Loss of Dnmt1 activity was shown to cause neurological abnormalities [96]. Loss of Dnmt1 in mouse embryonic cells leads to genome-wide demethylation, confirming the importance of DNMT1 in maintenance methylation [97]. Similarly, in mammalian systems, *DNMT1* or *UHRF1* knockout leads to dramatic global DNA hypomethylation [93,96,98]. In HCT116 colon cancer cells, loss of DNMT1 results in cell-cycle arrest and mitotic defects, leading to cell death [99]. Interestingly, DNMTs are often aberrantly expressed in several cancer types, and normal DNMT expression is essential for development in mammals [100]. Similarly, UHRF1 expression is also upregulated in cancer, resulting in global DNA hypomethylation [101,102]. Recent studies found the expression of two variant Dnmt1 isoforms: (1) Dnmt1o, which is expressed in the oocyte and pre-implantation embryo, and (2) Dnmt1p, which is expressed in pachytene spermatocytes [103,104]. Both Dnmt1o and Dnmt1p are expressed as a result of alternate usage of the first exon, and their loss results in abnormal DNA methylation of imprinted genes and lethality in the developing fetus [104].

### 2.2. DNMT3A

The *DNMT3A* locus expresses two isoforms, DNMT3A1 and DNMT3A2. *DNMT3A2* is expressed from an intronic promoter downstream of the *DNMT3A1* promoter, and lacks 223 amino acids in the *N*-terminal region. Both isoforms contain the PWWP domain for H3K36me3 interaction and the ADD domain for histone binding and transcriptional and epigenetic regulation. DNMT3A1 expression is maintained in differentiated cells and is localized to heterochromatic regions, where it interacts with DNA using the *N*-terminal domain [84,105]. In mice, undifferentiated cells, embryonic stem cells, and embryonal carcinoma cells, as well as the testis, ovary, thymus, and spleen, predominantly express Dnmt3a2 [106]. DNMT3A2 is the predominant DNMT3A isoform responsible for *de novo* DNA methylation in embryonic stem cells (Figure 1). DNMT3A2 expression is localized at euchromatic regions of the genome [106] where it is recruited by DNMT3L through its *C*-terminal catalytic domain. 

DNMT3A mutations result in dysregulation of its activity and aberrant DNA methylation patterns commonly reported during various developmental defects and hematological malignancies among others. Mouse *Dnmt3a* knockout (*Dnmt3a −/−*) nervous tissues resulted in retarded neuromuscular and motor activity, and subsequent premature death in adults; however, the embryos and birth rate were seemingly not affected [107]. DNMT3A mutations were also reported in overgrowth disorders in humans [108]. The symptoms of this syndrome include facial abnormality, pre- and postnatal overgrowth, and intellectual disability. DNMT3A mutations are also frequent in acute myeloid leukemia (AML) patients. The R882 (arginine) residue is the most frequent frame-shift mutation in AML patients; however, nonsense and splice site mutations were also reported. The R882H is a dominant negative mutation in the catalytic domain of DNMT3A, which affects the activity of the wild-type enzyme by inhibiting its homotetramerization [109]. 

### 2.3. DNMT3B

DNMT3B is expressed in more than 30 isoforms [110,111,112] with alternative splicing occurring in both the catalytic and regulatory domains. Full-length DNMT3B, DNMT3B1, is highly expressed in embryonic stem cells, but its expression decreases in somatic cells. DNMT3B3 is noted by alternative splicing of the catalytic domain, which was shown to affect its methyl-transfer function. DNMT3B3 is highly expressed in somatic cells (Figure 1) [113]. Interestingly, several catalytically inactive and active isoforms of DNMT3B were also discovered to act as accessory proteins in coordinating DNA methylation [113]. 

### 2.4. Dnmt3c

Recently, a duplicated copy of Dnmt3b, specific to mouse, termed Dnmt3c, was documented. Dnmt3c is truncated at the *N*-terminal domain and lacks the PWWP domain. Dnmt3c is exclusively expressed in male gonads and is involved in promoter DNA methylation of evolutionarily young retrotransposons. Dnmt3c resulted in male sterility, while females were unaffected [114]. 

### 2.5. DNMT3L

Dnmt3L acts as an accessory protein for Dnmt3a- and Dnmt3b-mediated *de novo* DNA methylation during gametogenesis and in embryos soon after fertilization [115]. Dnmt3L is expressed only in germ cells and embryonic stem cells, but not in somatic cells [116], and is involved in the regulation of repetitive elements and imprinting in germ cells [115,117]. Dnmt3L is predominantly expressed in the postnatal female germline along with Dnmt3a and Dnmt3b for the establishment of DNA methylation patterns. In contrast, Dnmt3L and Dnmt3a are expressed in male prenatal tissues [118,119,120]. 

The PHD domain of Dnmt3L interacts with genomic regions marked by unmethylated H3K4 [121]. This interaction also stabilizes and regulates the activity of Dnmt3a2 in mouse embryonic cells [122]. Mass spectroscopy of epitope-tagged Dnmt3L in mice revealed its interaction with Dnmt3a, Dnmt3b, and core histones, in that the *N*-terminal cysteine rich domain of Dnmt3L interacts with histone H3, and this interaction is inhibited in the presence of H3K4me3 [121]. In mouse embryonic stem cells, Dnmt3L regulates gene expression by promoting gene body DNA methylation of housekeeping genes. Contrary to popular belief, this group also showed that Dnmt3L negatively regulates DNA methylation at bivalent promoters (carrying both the active H3K4me3 histone mark and the repressive H3K27me3 histone mark) [123]. This is achieved through a competition between PRC2 and Dnmt3 to bind Dnmt3L at H3K27me3-occupied loci. [123]. More recently, these results were challenged by similar studies using *Dnmt3l* knockout cells from blastocyst-stage mouse embryos, where Dnmt3L was shown to contribute to Dnmt3a-dependent DNA methylation [122].

*DNMT3L* knockout human embryonal carcinoma cells resulted in apoptosis and suppression of growth [124]. In human fetal-derived kidney cells, co-expression of both DNMT3L and DNMT3A, but not DNMT3B, is required for DNA methylation of maternally imprinted genes [125]. The lack of DNMT3L expression in somatic cells raises an interesting point as to the unidentified accessory protein(s) during DNMT3A/DNMT3B-mediated DNA methylation in somatic cells. Recently, the catalytically inactive DNMT3B3 and DNMT3B4 isoforms and the catalytically active DNMT3B1 isoform were shown to acts as accessory proteins to DNMT3A/3B function [113,126]. 

## 3. Specific Roles of DNMT3B Isoforms

**Figure 2:**
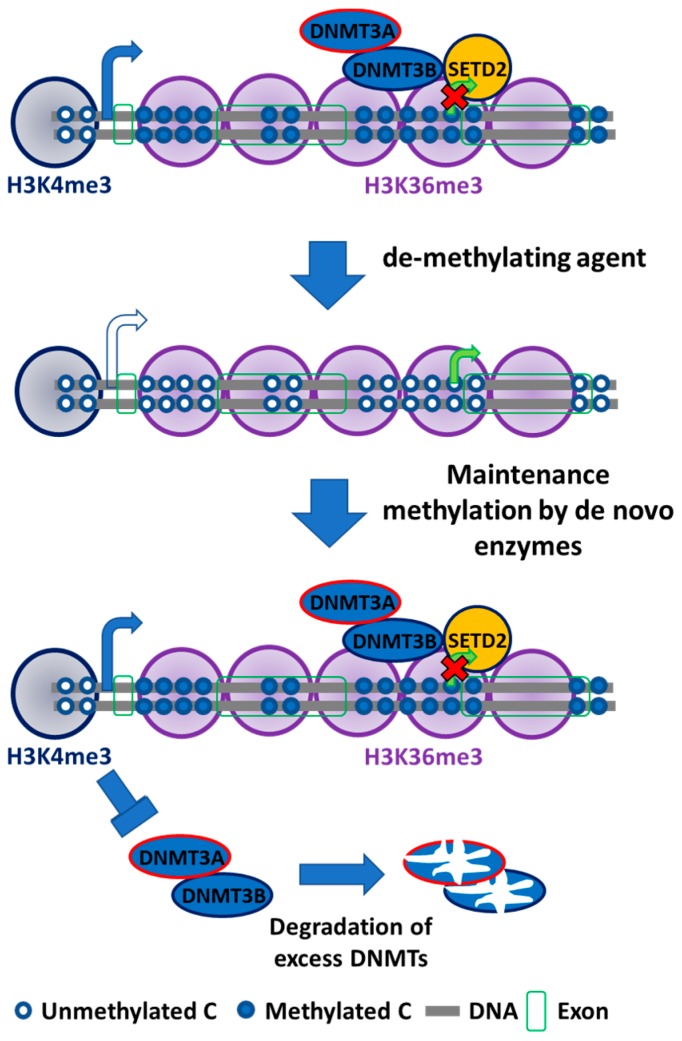
Mechanism of *de novo* DNA methyltransferases (DNMTs): DNMT3s localizes on methylated cytosine–guanine (CpG)-rich locus to maintain a uniform methylation pattern. DNMT3B targets gene body to methylate cytosine locus. DNMT3B acts as maintenance enzyme, complimenting the low fidelity of DNMT1 and as a *de novo* enzyme to mark new methylation patterns during differentiation and remethylation after treatment with demethylating agents. DNMT3B also interact with H3K36me3 to localize at these active expression marks. DNA methylation in the genic region is essential for efficient transcription, stability of splicing factors, stability of elongation factor, and to inhibit generation of spurious transcripts. DNMT3s localization at the promoter region is prevented by unmethylated cytosines and by H3K4me3. The free DNMT3s are unstable and are degraded.

In addition to their *de novo* methyltransferase function, DNMT3A and 3B have affinity toward methylated CpG sites in the genome. A recent study from our group demonstrated that DNA hypermethylation at transcribed gene regions is correlated with upregulated gene expression, and demethylating these hypermethylated regions via the DNA methylation inhibitor 5-aza-2’-deoxycytidine (5-aza-CdR) also downregulated the expression of these genes [48]. *DNMT3B* knockout cells resulted in reduced DNA remethylation after 5-aza-CdR treatment, highlighting its function in gene body DNA methylation, whereas *DNMT1* knockout had no effect on gene body DNA methylation. In addition, gene body DNA methylation was also correlated with the presence of H3K36me3 occupancy (Figure 2) [48,127], suggesting that DNMT3B may interact with H3K36me3 marks or histone-lysine N-methyltransferase SET domain containing 2 (SETD2), the enzyme responsible for placing these marks. To prove the role of H3K36me3 in recruiting DNMT3B, H3K36me3 levels were reduced by *SETD2* knockout. Depleted H3K36me3 was correlated with reduced DNMT3 co-localization. The unbound DNMT3A and DNMT3B are rapidly degraded through proteasomal and other unknown pathways [128]. In addition, the interaction between H3K36me3 and the PWWP domain of DNMT3B1 was also confirmed through in vitro experiments [78]. The chemical inhibition of RNA polymerase II did not affect DNMT3B binding to H3K36me3, showing its independence to transcription activity [78]. 

As the embryonic stem cells differentiate, DNMT3B1 expression (catalytically active) decreases and is replaced by increased expression of the catalytically inactive DNMT3B3 isoform. Since DNMT3B is responsible for transcribed gene body DNA methylation and DNA remethylation after 5-aza-CdR treatment, *DNMT3B* knockout cells fail to remethylate gene bodies after 5-aza-CdR treatment [113]. However, DNA remethylation can be restored in *DNMT3B* knockout cells if expression of DNMT3B1, DNMT3B3 (catalytically inactive), DNMT3B1M (mutated catalytic domain), or DNMT3L is restored. Furthermore, *DNMT3A* and *DNMT3B* double knockout cells including the inactive DNMT isoforms (DNMT3B1-M, DNMT3B3, and DNMT3L) fail to restore DNA methylation after 5-aza-CdR treatment. Thus, DNMT3B3 (without catalytic domain), DNMT3B1M (mutated catalytic domain), and DNMT 3L can remethylate CpG loci only in the presence of DNMT3A. Interestingly, gene body DNA remethylation is rescued after 5-aza-CdR treatment when the catalytically active DNMT3B1 isoform is exogenously expressed in *DNMT3A/DNMT3B* double knockout cells, demonstrating its involvement in gene body DNA methylation without DNMT3A involvement [113]. Thus, DNMT3B isoforms with or without its catalytic activity can act as accessory proteins for DNA methylation by recruiting DNMT3A to target regions [113,126]. This experiment also showed that DNMT3B1 may act as the main catalytic and accessory enzyme in gene body DNA methylation in somatic cells. It also showed that some catalytically inactive isoforms of DNMT3B may act as accessory proteins in *de novo* DNA methylation mechanisms. Somatic cells also express the catalytically active DNMT3B2 isoform that contains an intact catalytic domain but lacks exon 10 [74,129]. DNMT3B2 is expressed at low levels in somatic cells [130]. Thus, the characterization of all DNMT3B isoforms in terms of their unique and overlapping accessory roles may also explain the presence of aberrant DNA methylation patterns during tumorigenesis, and may highlight their role as a driving force for the *de novo* DNA methylation profiles in human cancers [113]. 

In accordance to the aforementioned absence of DNMT3B1 expression in somatic tissues, DNMT3B1 was found to be undetectable in normal lung tissue, while it was highly expressed in non-small-cell lung cancer tissues [111]. Recently, the catalytically inactive DNMT3B3 isoform was demonstrated to act as an accessory protein, much like DNMT3L, in cancer cells [113]. In addition, in vitro DNMT3B3 overexpression counteracted the stimulatory effect of DNMT3L by complexing with DNMT3L, resulting in reduced DNA methylation [126]. These results indicate that the aberrant expression of DNMTs in various forms of human cancer may disrupt DNA methylation profiles. 

**Figure 3:**
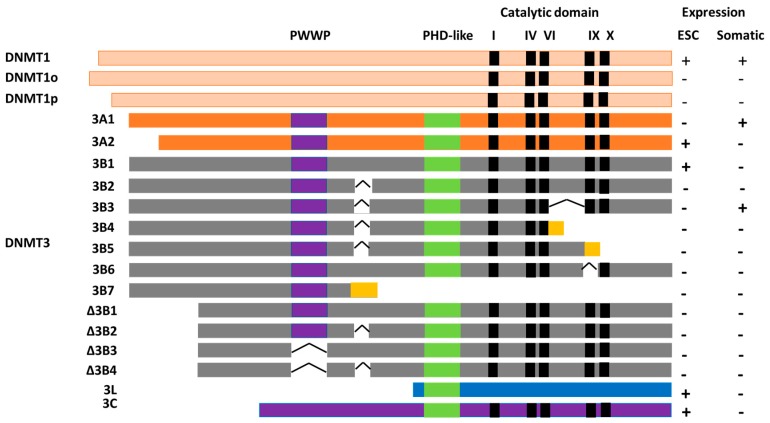
Schematic diagram showing DNA methyltransferase (DNMT) isoforms: DNMT3A (A1 and A2), DNMT3B (3B1, 3B2, 3B3, 3B4, 3B5, 3B6, 3B7, Δ3B1, Δ3B2, Δ3B3, and Δ3B4), and DNMT3L. DNMT3 consist of a PWWP domain (purple), a PHD-like domain (green), and a catalytic domain (black). The deletions are shown as black lines; frame-shift mutations are in yellow. The figure was modified and adapted from Duymich et al., 2016 [113]; Ostler et al., 2007 [110]; Gopalakrishnan et al., 2009 [112]; and Choi et al., 2011 [131]. ESC: Embryonic stem cells.

There are more than 30 DNMT3B isoforms [113,131,132] in human and mouse cells. The domain structures of the most common isoforms are presented in Figure 3. Most of these isoforms are aberrantly expressed in cancer cells but not in normal cells [110]. These isoforms are characterized by exon deletion, premature 3’ termination, alternatively spliced exons, fusion of intronic sequences, and missing 5’ exons. These modifications affect their catalytic activity, as well as their cellular localization and interactions with DNA and the nucleosome, [133]. The specific functional roles of most isoforms are not known. However, the roles of some isoforms were reported and are discussed here. Deletion of part of or the entire catalytic domain, located in the *C*-terminal region of DNMT3B isoforms, is characteristic of the DNMT3B3, 3B4, 3B5, 3B6, and 3B7 isoforms, all of which are catalytically inactive. DNMT3B4, B5, and B7 have an additional frame-shift mutation that introduces an early stop codon, thereby causing inactivity of the enzymes. Interestingly DNMT3B4 failed to remethylate DNA in the presence of DNMT3A which suggested that the loss of catalytic domain of DNMT3B4 also affected its ability to act as an accessory protein [113], this may suggest a failure of DNMT3B4 to bind to nucleosome. The specific functions and mechanisms of action of these novel exons coded by these frame shifts remain unknown. Deletions in the DNMT3B *C*-terminal region may affect its interaction with DNMT3L, as in vitro studies showed that the DNMT3B1 *C*-terminal catalytic domain interacts with DNMT3L [133]. 

Overexpression of the catalytically inactive DNMT3B isoforms is associated with aberrant DNA methylation patterns. In hepatocellular carcinoma, DNMT3B4 overexpression is associated with hypomethylation of pericentromeric satellite regions [134]. This might be due to the competition of DNMT3B4 with the catalytically active DNMT3B3 isoform that is expressed in normal liver cells [134]. More recently, the sequestering nature of DNMT3B4 toward DNMT3A/3B was also reported, and may be a contributing factor of pericentromeric satellite DNA hypomethylation and genomic instability [126]. The DNMT3B7 isoform may also contribute aberrant alteration in DNA methylation and gene expression in cancer [110], as it is widely expressed in various hematopoietic and solid tumor cell lines. In addition, DNMT3B7 was also found to be highly expressed in differentiated ganglioneuroblastomas as compared to the undifferentiated neuroblastomas. DNMT3B7 expression in neuroblastoma cells correlates with altered gene expression and tumor growth inhibition [135]. 

DNMT3B isoforms lacking the *N*-terminal domain, termed ΔDNMT3B isoforms, first reported by Wang et al., are predominantly expressed in non-small-cell lung cancer (NSCLC) [111]. Seven variants of those isoforms were reported and, interestingly, ΔDNMT3B1, ΔDNMT3B2, and ΔDNMT3B4 expression was correlated with *CDKN2A (p16)* and *RASSF1A* promoter DNA methylation in tumor cells [136,137]. The *N*-terminal region of DNMT3B is essential for strong nucleosome binding, as shown by experiments on Δisoforms of DNMT3B2 (ΔDNMT3B2) that lack the *N*-terminal domain, as well as ΔDNMT3B4 that lacks both the *N*-terminal and the PWWP domains [79,128]. The weak association of ΔDNMT3B with nucleosomes eventually results in decreased DNA methylation [113,133]. In vitro studies in which the PHD-like or PWWP domains and the region joining the PWWP and PHD domains were deleted did not have any effect on the interaction between DNMT3B and DNMT3L, whereas deletion of the *N*-terminal region and a portion of the PWWP domain inhibited interaction with DNMT3B1 [133]. The PWWP domains of DNMT3s are involved in interactions with DNA, and their absence reduces DNMT3 binding affinity to DNA [138]. Furthermore, the PWWP domain modulates interaction between epigenetic marks, together with the histones and DNA modifiers or readers [139]. These findings indicated that aberrantly expressed DNMT3B isoforms in cancer may have specific DNA target sequences, which was later confirmed [131].

## 4. Cooperativity Between DNA Methyltransferases 

Established DNA methylation profiles are maintained during each cell division; however, the presence of hemimethylated sequences was reported in a very small proportion of single-copy genes and repetitive elements [76]. This proportion increased in *Dnmt3a/Dnmt3b* double knockout cells, indicating cooperativity between DNMTs [77]. However, Dnmt1 does not have an absolute fidelity, and proofreads to methylate unmethylated DNA quickly after cellular replication and division [76,95]. In vitro experiments implicated the proof-reading activity of DNMT1 in *de novo* DNA methylation mediated by DNMT3A. DNA methylation was observed to be five times higher in the presence of DNMT3A and DNMT1 [75]. The cooperativity between Dnmt3s and Dnmt1 was later also confirmed by experiments in cultured mouse embryonic stem cells [140]. An exception exists in the adult intestinal cells, where Dnmt1 or Dnmt3B alone is sufficient to maintain DNA methylation in dividing cells. *Dnmt1* knockout correlates with induced Dnmt3B expression, and the ablation of both Dnmts resulted in a genome-wide DNA hypomethylation, genomic instability, and increased apoptosis and lethality [141]. 

## 5. Future Perspectives

Since the initial discovery of epigenetic factors as drivers for diseases including cancer, many tools and strategies were developed for studying the mechanisms contributing to diseases. This led to a seemingly large number of tools useful for the treatment of epigenetic diseases. The discovery of 5-azacytidine (5-aza-CR) and 5-aza-CdR as DNA demethylation agents [142] shows promise in the treatment of several forms of human cancer. Both drugs were approved by the Food and Drug Administration for the treatment of myelodysplastic syndrome (MDS) and acute myeloid leukemia (AML) [143,144,145]. A more comprehensive list of drugs for targeting the epigenome is provided in Reference [146]. DNMT accessory proteins may also be efficacious targets for epigenetic therapies, as are histone-modifying enzymes that regulate chromatin structure [147]. The development of plasmid constructs carrying a fusion of an altered clustered regularly interspaced short palindromic repeats (CRISPR)–Cas9 system with a DNMT/TET construct may one day be used in curing diseases related to gene imprinting or cancer [148,149,150]. Nonetheless, unveiling epigenetic enzymes and their mechanisms of action holds tremendous promise for therapeutics and our understanding of cellular processes.
